# Shikimate and Phenylalanine Biosynthesis in the Green Lineage

**DOI:** 10.3389/fpls.2013.00062

**Published:** 2013-03-27

**Authors:** Takayuki Tohge, Mutsumi Watanabe, Rainer Hoefgen, Alisdair R. Fernie

**Affiliations:** ^1^Max-Planck-Institute of Molecular Plant PhysiologyPotsdam-Golm, Germany

**Keywords:** shikimate pathway, aromatic amino biosynthesis, evolution, gene copy number, gene duplication, plant secondary phenolic metabolite

## Abstract

The shikimate pathway provides carbon skeletons for the aromatic amino acids l-tryptophan, l-phenylalanine, and l-tyrosine. It is a high flux bearing pathway and it has been estimated that greater than 30% of all fixed carbon is directed through this pathway. These combined pathways have been subjected to considerable research attention due to the fact that mammals are unable to synthesize these amino acids and the fact that one of the enzymes of the shikimate pathway is a very effective herbicide target. However, in addition to these characteristics these pathways additionally provide important precursors for a wide range of important secondary metabolites including chlorogenic acid, alkaloids, glucosinolates, auxin, tannins, suberin, lignin and lignan, tocopherols, and betalains. Here we review the shikimate pathway of the green lineage and compare and contrast its evolution and ubiquity with that of the more specialized phenylpropanoid metabolism which this essential pathway fuels.

## Introduction

The shikimate pathway is closely interlinked with those of the aromatic amino acids (L-tryptophan, l-phenylalanine, and L-tyrosine) and in land plants bears very high fluxes with estimates of the amount of fixed carbon passing through the pathway varying between 20 and 50% (Weiss, 1986; Corea et al., 2012; Maeda and Dudareva, 2012). Considerable research focus has been placed on this pathway since the aromatic amino acids are not produced by humans and monogastric livestock and are therefore an important dietary component (Tzin and Galili, 2010). Furthermore, one of the enzymes of the pathway – 5-*enol*pyruvalshikimate-3-phosphate synthase (EPSP) – is one of the most widely employed herbicide target sites (see, Duke and Powles, 2008). Moreover, as we have recently described, plant phenolic secondary metabolites and their precursors are synthesized via the pathway of shikimate biosynthesis and its numerous branchpoints (Tohge et al., 2013). The shikimate pathway is highly conserved being found in fungi, bacteria, and plant species wherein it operates in the biosynthesis of not just the three aromatic amino acids described above but also of innumerable aromatic secondary metabolites such as alkaloids, flavonoids, lignins, and aromatic antibiotics. Many of these compounds are bioactive as well as playing important roles in plant defense against biotic and abiotic stresses and environmental interactions (Hamberger et al., 2006; Maeda and Dudareva, 2012), and as such are highly physiologically important. It is estimated that under normal conditions as much as 20% of the total fixed carbon flows through to shikimate pathway (Ni et al., 1996), with greater carbon flow through the pathway under times of plant stress or rapid growth (Corea et al., 2012). Given its importance it is perhaps not surprising that all members of biosynthetic genes and corresponding enzymes involved in shikimate pathway have been characterized in model plants such as *Arabidopsis*. Cross-species comparison of the shikimate biosynthetic enzymes has revealed that they share sequence similarity, divergent evolution, and commonality in reaction mechanisms (Dosselaere and Vanderleyden, 2001). However, all other species vary considerably from fungi which has evolved a complex system with a single pentafunctional polypeptide known as the AroM complex which performs five consecutive reactions (Lumsden and Coggins, 1977; Duncan et al., 1987). In this review we will summarize current knowledge concerning the genetic nature of this pathway focusing on cross-species comparisons bridging a wide range of species including algae (*Chlamydomonas reinhardtii*, *Volvox carteri*, *Micromonas* sp., *Ostreococcus tauri, Ostreococcus lucimarinus*), moss (*Selaginella moellendorffii*, *Physcomitrella patens*), monocots (*Sorghum bicolor*, *Zea mays*, *Brachypodium distachyon*, *Oryza sativa* ssp. *japonica* and *Oryza sativa* ssp. *indica*), and dicots (*Vitis vinifera*, *Theobroma cacao*, *Carica papaya*, *Arabidopsis thaliana*, *Arabidopsis lyrata*, *Populus trichocarpa*, *Ricinus communis, Manihot esculenta, Malus domestica*, *Fragaria vesca*, *Glycine max*, *Lotus japonicus*, *Medicago truncatula*) species (Table 1). Finally, we compare and contrast the evolution of this pathway with that of the more specialized pathways of phenylpropanoid biosynthesis.

**Table 1:** **Summary of the species used in the study**.

	Species name	ID	Common name	Classification	Species
1	*Chlamydomonas reinhardtii*	CR	Green algae	Chlorophyte	Chlamydomonadaceae
2	*Volvox carteri*	VC	Algae	Chlorophyte	Volvoceae
3	*Micromonas* sp. *RCC299*	MRC	Micromonas	Chlorophyta	Prasinophyceae
4	*Ostreococcus tauri*	OT	Microalgae	Prasinophyte	Prasinophyceae
5	*Ostreococcus lucimarinus*	OL	Microalgae	Prasinophyte	Prasinophyceae
6	*Selaginella moellendorffii*	SM	Spike moss	Lycophytes	Selaginellaceae
7	*Physcomitrella patens*	PP	Moss	Lycophytes	Funariaceae
8	*Sorghum bicolor*	SB	Sorghum	Monocot	Poaceae
9	*Zea mays*	ZM	Corn	Monocot	Poaceae
10	*Brachypodium distachyon*	BD	Purple false brome	Monocot	Poaceae
11	*Oryza sativa* ssp. *japonica*	OS	Japonica rice	Monocot	Poaceae
12	*Oryza sativa* ssp*. indica*	OSI	Indica rice	Monocot	Poaceae
13	*Vitis vinifera*	VV	Grapevine	Dicot	Vitaceae
14	*Theobroma cacao*	TC	Cacao	Dicot	Malvaceae
15	*Carica papaya*	CP	Papaya	Dicot	Caricaceae
16	*Arabidopsis thaliana*	AT	Arabidopsis	Dicot	Brassicaceae
17	*Arabidopsis lyrata*	AL	Lyrata	Dicot	Brassicaceae
18	*Populus trichocarpa*	PT	Poplar	Dicot	Salicaceae
19	*Ricinus communis*	RC	Castor oil plant	Dicot	Euphorbiaceae
20	*Manihot esculenta*	ME	Cassava	Dicot	Euphorbiaceae
21	*Malus domestica*	MD	Apple	Dicot	Rosaceae
22	*Fragaria vesca*	FV	Strawberry	Dicot	Rosaceae
23	*Glycine max*	GM	Soybean	Dicot	Fabaceae
24	*Lotus japonicus*	LJ	Lotus	Dicot	Fabaceae
25	*Medicago truncatula*	MT	Medicago	Dicot	Fabaceae

*Coding genes is estimated by Plaza (http://bioinformatics.psb.ugent.be/plaza/). Relationships among the species considered are presented on the Plaza website (http://bioinformatics.psb.ugent.be/plaza/)*.

## Shikimate Biosynthesis and Phenylalanine Derived Secondary Metabolism in Plants

**Figure 1:**
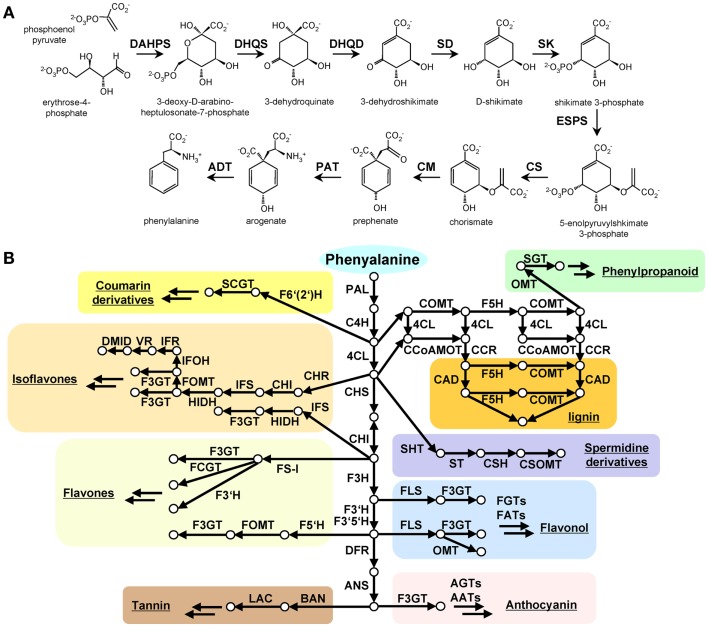
**The shikimate and *phenylalanine derived secondary metabolite* biosynthesis in plants**. **(A)** Shikimate biosynthesis starting from phosphoenolpyruvate (PEP) and D-erythrose 4-phosphate is described with characterized genes and reported intermediate metabolites. **(B)** phenylalanine derived major phenolic secondary mebolite biosynthesis in the green lineage. Arrow indicates enzymatic reaction, circle indicates metabolite. Abbreviation: DAHPS, 3-deoxy-D-arabino-heptulosonate 7-phosphate synthase; DQS, 3-dehydroquinate synthase; DHQD/SD, 3-dehydroquinate dehydratase; SK, shikimate kinase; ESPS, 3-phosphoshikimate 1-carboxyvinyltransferase; CS, chorismate synthase; CM, chorismate mutase; PAT, prephenate aminotransferase; ADT, arogenate dehydratase. PAL, phenylalanine ammonia-lyase; C4H, cinnamate-4-hydroxylase; 4CL, 4-coumarate CoA ligase; CAD, cinnamoyl-alcohol dehydrogenase; F5H, ferulate 5-hydroxylase; C3H, coumarate 3-hydroxylase; ALDH, aldehyde dehydrogenase; CCR, cinnamoyl-CoA reductase; HCT, hydroxycinnamoyl-Coenzyme A shikimate/quinate hydroxycinnamoyltransferase; CCoAOMT, caffeoyl/CoA-3-*O*-metheltransferase; CHS, chalcone synthese; CHI, chalcone isomerase; F3H, flavanone 3-hydroxylase; F3′H, flavonoid-3′-hydroxylase; F3GT, flavonoid-3-*O*-glycosyltransferase; FS, flavone synthase; FOMT, flavonoid *O*-methyltransferase; FCGT, flavone-*C*-glycosyltransferase; FLS, flavonol synthese; F3GT, flavonoid-3-*O*-glycosyltransferase; DFR, dihydroflavonol reductase; ANS, Anthocyanidin synthese; AGT, Flavonoid-*O*-glycosyltransferase; AAT, anthocyanin acyltransferase; BAN, oxidoreductase|dihydroflavonol reductase like; LAC, laccase.

Given that phenolic secondary metabolites which are derived from phenylalanine via shikimate biosynthesis are widely distributed in plants and other eukaryotes, genes encoding shikimate biosynthetic enzymes are generally highly conserved in nature. Eight and two reactions are involved in shikimate and phenylalanine biosynthesis, respectively. Both members of all gene families and the corresponding biosynthetic enzymes involved in these pathways have been characterized in model plants such as *Arabidopsis* (Figure 1A). In contrast, phenolic secondary metabolites derived from phenylalanine display considerable species-specific distribution with the phenolic secondary metabolites have been found in plant kingdom such as coumarin derivatives, monolignal, lignin, spermidin derivatives, flavonoid, tannin being present in specific families within the green lineage (Figure 1B). This diversity has arisen by the action of diverse evolutionary strategies for example gene duplication and *cis*-regulatory evolution in order to adapt to prevailing environmental conditions. Given their species-specific distribution, the genes involved in plant phenolic secondary metabolism such as phenylammonia-lyase (PAL), polyketide synthase (PKS), 2-oxoglutarate-dependent deoxygenases (2ODDs), and UDP-glycosyltransferases (UGTs) are frequently used as case studies of plant evolution (Tohge et al., 2013). Despite the fact that shikimate-phenylalanine biosynthetic genes are well conserved in all species including algae species, phenolic secondary metabolism related orthologous genes were not detected in all algae species (Table 2, Tohge et al., 2013). This result suggests a considerably more ancient origin of the shikimate-phenylalanine pathways. In the next sections, we will discuss the evolution of shikimate-phenylalanine pathways focusing on cross-species comparisons for each gene encoding on of the constituent enzymes of either pathway.

**Table 2:** **Shikimate and phenylalanine biosynthetic genes and homologs in each species with/without tandem duplicated genes**.

No. ID	1 CR	3 MRCC299	4 OT	8 SB	9 ZM	10 BD	11 OS	12 OSindica
DHS	Cr17g06460	Mrcc02g07760	Ot06g03510	Sb01g028770	Zm02g39200	Bd1g21330	Os03g27230	Osi07g35030
				Sb01G033590	Zm04g31550	Bd1g60750	Os07g42960	Osi08g36090
				Sb02G039660	Zm05g06990	**Bd3g33650**	Os08g37790	Osi10g31830
				Sb07G029080		**Bd3g38670**	Os10g41480	

DQS	Cr08g02240	Mrcc01g05190	Ot05g01830	Sb02G031240	Zm02g34320	Bd4g36507	Os09g36800	Osi09g29080

DHQD	Cr08g04550	Mrcc01g03580	Ot12g02660	Sb08G016970	Zm03g17940	Bd4g05897	Os12g34874	Osi12g23310
					Zm10g05140			

SK	Cr10g04010	Mrcc13g02500	Ot14g03180	Sb06G030260	Zm02g02970	Bd3g59237	Os04g54800	Osi02g49680
					Zm04g27840	Bd5g23460	
					Zm05g40530	

SKL1				Sb08G018630	Zm01g26660	Bd2g03680	Os01g01302	

SKL2		Mrcc02g03490	Ot07g01450	Sb01G027930	Zm01g22640	Bd3g34245	Os10g42700	

ESPS	Cr03g06830	Mrcc13g01100	Ot14g02430	Sb10G002230	Zm09g05500	Bd1g51660	Os06g04280	Osi06g03190

CS	Cr01g12390	Mrcc05g01430	Ot02g06020	Sb01G040790	Zm01g10020	Bd1g67790	Os03g14990	Osi03g13340
					Zm09g24540	

CM	Cr03g01600	Mrcc08g05060	Ot08g02860	Sb03G035460	Zm03g31000	Bd2g50800	Os01g55870	Osi01g52850
				Sb04G005480	Zm05g21270	Bd3g06050	Os02g08410	Osi02g08160
					**Zm08g34320**		Os12g38900	
					**Zm08g34330**	

PAT	Cr02g15900	Mrcc06g00860	Ot16g00690	Sb03G041180	Zm03g25600	Bd2g24300	Os01g65090	Osi01g61700
				Sb09G021360	Zm08g15210	Bd2g56330	

ADT	Cr06g02760	Mrcc01g05870	Ot01g01250	Sb01G038740	Zm01g12020	**Bd5g09020**	Os04g33390	Osi03g16350
				Sb06G015310	Zm02g16320	**Bd5g09030**	Os03g17730	Osi04g25440
					Zm10g16000	Bd1g16517	Os07g49390	Osi07g41390
						Bd1g65800	

No. ID	13 VV	14 TC	16 AT	17 AL	18 PT	21 MD	22 FV	23 GM	24 LJ	25 MT
DHS	Vv00g09200	Tc01g008590	At1g22410	Al1g23930	Pt01g14860	Md00g000730	Fv0g22320	Gm02g37080	Lj1g002520	Mt2g009080
	Vv00g17890	Tc01g012940	At4g33510	Al7g02250	Pt02g09760	Md00g361080	Fv5g19610	Gm06g10670		Mt5g064500
	Vv18g03830	Tc02g011250	At4g39980	Al7g07720	Pt05g07260	Md01g001320		Gm14g35370	
		Tc03g024120			Pt05g16320	Md04g002280		Gm15g06020	
		Tc08g008780			Pt07g04970	Md05g021570	
						Md05g025390	
						Md10g003880	
						Md11g021260	

DQS	Vv04g00350	Tc01g001360	At5g66120	Al8g34560	Pt05g11110	Md00g089850	Fv1g13270	Gm01g36890	Lj2g022420	Mt5g022580
								Gm11g08350	

DHQD	Vv05g03610	Tc04g027300	At3g06350	Al3g06450	Pt10g01690	Md00g196450	Fv1g19500	Gm01g20760	Lj4g005930	Mt4g090620
	**Vv14g04450**	**Tc05g024340**			Pt13g02880	Md00g199470	**Fv6g07230**	Gm20g37400	
	**Vv14g04460**	**Tc05g024370**				Md00g208810	**Fv6g07240**	
						**Md01g014110**	
						**Md01g014130**	
						Md04g017400	
						Md15g026460	

SK	Vv00g22160	Tc01g010070	At2g21940	Al4g01190	Pt02g06000	Md00g396950	Fv6g01580	**Gm04g39700**	Lj1g014890	
	Vv07g06350		At4g39540	Al7g01530	Pt05g08460	Md02g009820		**Gm04g39710**	
					Pt07g06400			Gm05g31730	
								Gm08g14980	

SKL1	Vv14g14000	Tc04g004710	At3g26900	Al5g05650	Pt17g08780		Fv6g51520	Gm02g08050	Lj1g008480	
								Gm16g27060	

SKL2	Vv02g01940	Tc03g029930	At2g35500	Al4g20870	Pt03g08570	Md00g061570	Fv0g29740	Gm01g01890	Lj3g020970	Mt1g009450
						Md00g432830	Fv2g18080		Lj3g020980	Mt5g029550
						Md06g002680	

ESPS	**Vv15g09330**	Tc01g037810	At1g48860	Al1g42610	Pt02g14550	Md00g030870	Fv7g11420	Gm01g33660	Lj3g025840	Mt4g024620
	**Vv15g09350**		At2g45300	Al4g33160	Pt14g06200	Md00g271560		Gm03g03190	

CS	Vv06g05280	Tc10g005370	At1g48850	Al1g42550	Pt08g03850	Md00g355380	**Fv4g18660**	Gm10g35560	Lj0g038950	**Mt1g095160**
	Vv13g03240			Al3g19880	Pt10g21700	Md01g008950	**Fv4g18670**	Gm20g31980	Lj0g284550	**Mt1g095240**
						Md08g005430	**Fv7g23950**			**Mt1g095250**
							**Fv7g24040**	
CM	Vv01g02110	Tc02g032570	At1g69370	Al2g17620	Pt10g15830	Md00g250450	Fv0g04690	Gm13g05830	**Lj5g005890**	Mt1g013820
	Vv04g13080	Tc04g009770	At3g29200	Al5g08750	Pt17g12090	Md00g329990	Fv2g52320	Gm14g11870	**Lj5g005900**	Mt5g043210
	Vv14g02700	Tc09g001490	At5g10870	Al6g10610	Pt18g02330	Md01g020010	Fv6g43680	Gm17g33940	
						Md16g003330		Gm19g03290	
						Md17g004580	

PAT	Vv07g05790	Tc01g009420	At2g22250	Al4g01710	Pt05g07910	Md00g135490	Fv6g00440	Gm05g31490	Lj6g003720	Mt8g091280
	Vv18g03130				Pt07g05690	Md00g246930		Gm08g14720	
						Md00g304630		**Gm11g36190**	
								**Gm11g36200**	

ADT	Vv06g04790	Tc02g034990	At1g08250	Al1g12100	Pt00g13690	**Md00g099570**	Fv3g01120	Gm11g15750	Lj3g029800	Mt2g088130
	Vv10g00970	Tc06g019290	At1g11790	Al3g08080	Pt04g01150	**Md00g099580**	Fv3g16180	Gm11g19430	Lj4g001780	Mt4g055310
	Vv12g10860	Tc09g026620	At2g27820	Al4g12300	Pt04g18820	Md00g456520	Fv3g29940	Gm12g07720		Mt4g061070
		Tc09g028840	At3g07630	Al5g12520	Pt08g19820	Md05g001400		Gm12g09050		Mt4g132250
			At3g44720	Al6g22310	Pt09g14910	Md15g019040		Gm12g30660	
			At5g22630					Gm12g31940	
								Gm17g01610	

*Orthologous genes were estimated by BLAST search in Plaza website. Bold indicates tandem gene duplication*.

## 3-Deoxy-D-Arabino-Heptulosonate 7-Phosphate Synthase

**Figure 2:**
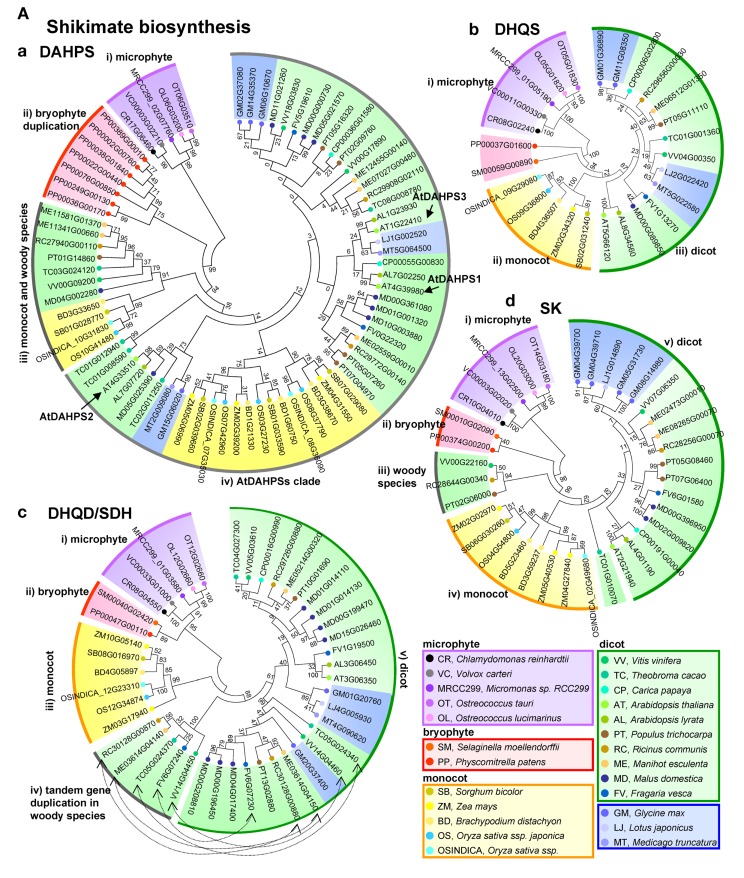

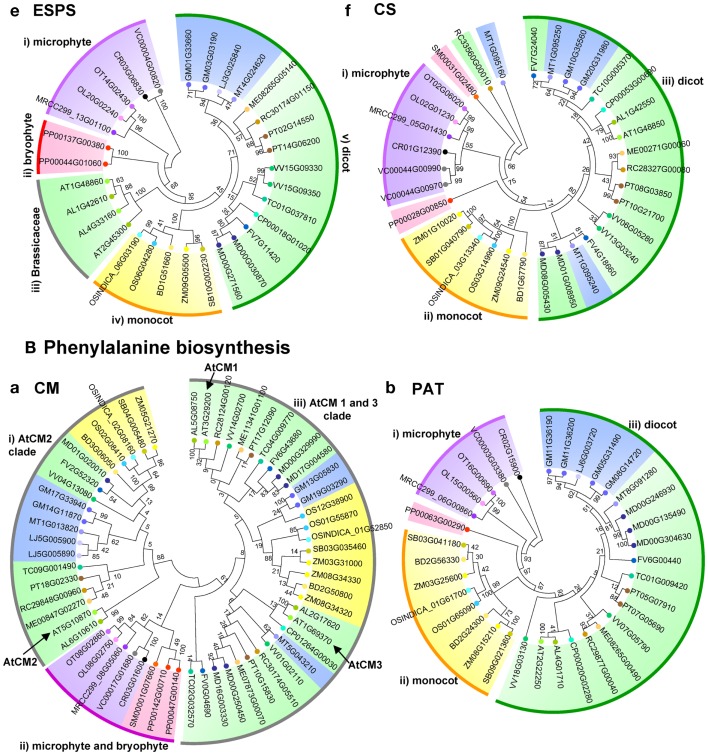
**Phylogenetic tree analysis of shikimate and phenylalanine biosynthetic genes in 25 species**. Amino acid sequence phylogenetic trees of **(A)** shikimate pathway: (a), DAHPS, (b) DHS, (c) DHQD/SD, (d) SK, (e) ESPS, and (f) CS, **(B)** phenylalanine related genes, (a) CM and (b) PAT. Amino acid sequences of shikimate biosynthetic genes are obtained from Plaza database (http://bioinformatics.psb.ugent.be/plaza/). Relationships among the species considered are presented on the Plaza website. The phylogenetic tree was constructed with the aligned protein sequences by MEGA (version 5.10; http://www.megasoftware.net/; Kumar et al., 2004) using the neighbor-joining method with the following parameters: Poisson correction, complete deletion, and bootstrap (1000 replicates, random seed). The protein sequences were aligned by Plaza. Values on the branches indicate bootstrap support in percentages.

The first enzymatic step of the shikimate pathway, 3-deoxy-D-arabino-heptulosonate 7-phosphate synthase (DAHPS), catalyzes an aldol condensation of phospho*enol*pyruvate (PEP), and D-erythrose 4-phosphate (E4P) to produce 3-deoxy-D-arabino-heptulosonate 7-phosphate (DAHP) (Figure 1). According to their protein structure, DAHPSs can be clustered into two distinct homology classes. The microbe derived class I DAHPS contain a bifunctional chorismate mutase (CM)-DAHPS domains, for that reason microbial DAHPSs, for example, *E. coli* (AroF, G, and H) and *S. cerevisiae* (Aro3 and 4), are classified as class I DAHPSs. By contrast, class II DAHPS were previously thought to be present only in plant species, but have subsequently been reported in certain microbes such as *Streptomyces coelicolor*, *Streptomyces rimosus*, and *Neurospora crassa* (Bentley, 1990; Maeda and Dudareva, 2012). The DAHPS (*AroA*) and CM (AroQ) activities of *B. subtilis* DAHPS are, however, separated by domain truncation. Detailed sequence structure analysis of the bacterial *AroA* and AroQ families, enzymatic studies with the full-length protein and the truncated domains of *AroA* and AroQ of *B. subtilis*, and comparison with fusion proteins of *Porphyromonas gingivalis* in which the AroQ domain was fused to the C terminus of *AroA*, suggest that “feedback regulation” may indeed be the evolutionary link between the two classes which are evolved from primitive unregulated member of class II DAHPS (Wu and Woodard, 2006). Class II plant DAHPSs have been reported from carrot roots (Suzich et al., 1985) and potato cell culture (Pinto et al., 1986; Herrmann and Weaver, 1999). DAHPS is encoded by three genes in the *Arabidopsis* genome (AtDAHPS1, AT4G39980; AtDAHPS2, At4g33510; AtDAHPS3, At1g22410). Orthologous gene search queries using the *Arabidopsis* DAHPSs, revealed a single gene in algae species (*Chlamydomonas reinhardtii*, *Volvox carteri*, *Micromonas* sp., and *Ostreococcus tauri*) and *Lotus japonica* but two to eight isoforms in other higher plant species (Table 2). AtDAHPS1-type and AtDAHPS2 type genes display differential expression in *Arabidopsis thaliana*, *Solanum lycopersicum*, and *Solanum tuberosum* (Maeda and Dudareva, 2012). AtDAHPS1-type genes, which are additionally subject to redox regulation by the ferredoxin-thioredoxin system, exhibit significant induction by wounding and pathogen infection (Keith et al., 1991; Gorlach et al., 1995; Maeda and Dudareva, 2012), whereas AtDAHPS2 type genes display constitutive expression (Gorlach et al., 1995). A phylogenetic analysis of DAHPS genes reveals four major clades, (i) a microphyte clade, (ii) a bryophyte duplication clade, (iii) monocot and dicot woody species clade, (iv) a AtDAHPSs clade (Figure 2Aa). Furthermore, major clade iv has four sub-groups, (iv-a) AtDAHPS2 group, (iv-b) monocot, (iv-c) AtDAHPS1 group and (iv-d) AtDAHP3 group. This result indicates that the constitutively expressed AtDAHPS1 and the stress responsive AtDAHPS 3 type genes display well conserved sequence between species (clade iv-c and iv-d), whereas the second constitutively expressed AtDAHPS2 type genes are clearly separated between monocot and dicot species (clade iv-a).

## 3-Dehydroquinate Synthase

The second step of the shikimate pathway is catalyzed by 3-dehydroquinate synthase (DHQS), an enzyme which promotes the intramolecular exchange of the DAHP ring oxygen with carbon 7 to convert DAHP into 3-dehydroquinate. Unlike the fungal situation detailed above, the plant DHQS gene is monofunctional and only found as a single copy in all species with the exception *Glycine max* which harbors two genes in its genome (Figure 2Ab). Phylogenetic analysis of DHQS genes reveals three major clades consisting of (i) microphyte (ii) bryophyte, (iii) monocot, (iv) Brassicaceae, and (v) dicot species. Intriguingly, by contrast to other shikimate biosynthetic genes, gene expression of DHQS gene is not well correlated to phenylpropanoid production in *Arabidopsis* (Hamberger et al., 2006).

## 3-Dehydroquinate Dehydratase/Shikimate Dehydrogenase

3-Deoxy-d-arabino-heptulosonate 7-phosphate is converted to 3-dehydroquinate by the bifunctional enzyme 3-dehydroquinate dehydratase/shikimate dehydrogenase (DHQD/SD), which catalyzes firstly the dehydration of DAHP to 3-dehydroshikimate and consequently the reversible reduction of this intermediate to shikimate using NADPH as co-factor. DHQD/SD exists in three forms; bacterial specific class I shikimate dehydrogenases (AroE type), class II shikimate/quinate dehydrogenases (YdiB type), and class III of shikimate dehydrogenase-like (SHD-l type) (Michel et al., 2003; Singh et al., 2005). In plants class IV, enzymatic activity of DHQD is 10 times higher than SD activity indicating that the amount of 3-dehydroshikimate will be more than sufficient to support flux through the shikimate pathway (Fiedler and Schultz, 1985). This bifunctional enzyme plays an important role in regulating metabolism of several phenolic secondary metabolic pathways (Bentley, 1990; Ding et al., 2007). In general, seed plants contain a single DHQD/SD gene which contains a sequence encoding a plastic transit peptide in their genome (Maeda et al., 2011, Table 2). However, an exception to this statement is *Nicotiana tabacum* which contains two genes in its genome. Intriguingly, silencing of NtDHD/SHD-1 results strong growth inhibition and reduction of the level of aromatic amino acids, chlorogenic acid, and lignin contents (Ding et al., 2007), however, a second cytosolic isoform can compensate for the production of shikimate but not at the phenotypic level. On a more general basis phylogenetic analysis reveals that microphytes also contain a low number of DHQD/SD genes (between one and two), whilst clear separation between (i) the microphyte clade, (ii) bryophyte clade, (iii) monocot clade, (iv) woody species-specific tandem gene duplication clade, and (v) dicot clades could be observed (Figure 2Ac; Table 2). Interestingly, the observation of the woody species-specific tandem gene duplication clade suggests that these species evolved after DHQD/SD gene duplication. The cytosolic localization of NtDHD/SHD-2 is intriguing since the presence of DAHP synthase, ESPS synthase and CM isoforms lacking N-terminal plastid targeting sequences has been reported (d’Amato, 1984; Mousdale and Coggins, 1985; Ganson et al., 1986). Furthermore, the findings that both ESPS synthase and shikimate kinase (SK) are active even when they retain their target sequences (Dellacioppa et al., 1986; Schmid et al., 1992) suggests that they could also potentially be constituents of a cytosolic pathway. Finally, experiments in which isolated and highly pure mitochondria were supplied with ^13^C labeled glucose to investigate the binding of the cytosolic isoforms of glycolysis (Giege et al., 2003) also revealed ^13^C enrichment in shikimate (Sweetlove and Fernie, 2013), indicating that a full cytosolic pathway is likely also in this species.

## Shikimate Kinase

The fifth reaction of the shikimate pathway is catalyzed by SK which catalyzes the ATP-dependent phosphorylation of shikimate to shikimate 3-phospate (S3P). *E. coli* has two SKs, one of class I (AroL type) and one of II (AroK type) which share only 30% sequence identity (Griffin and Gasson, 1995; Whipp and Pittard, 1995; Herrmann and Weaver, 1999). In plants, different numbers of SK isoforms are found in several species; only one in green algae, lycophytes, and bryophytes but between one and three in monocot and dicot plants (Table 2). A phylogenetic analysis of SK genes presents five major clades consisting of (i) microphyte, (ii) bryophyte, (iii) dicot woody species-specific clade, (iv) monocot clade, and (v) dicot species clade (Figure 2Ad). Anaylsis of the SK protein of *Spinacia olerancea* revealed that it was modulated by energy status and is therefore similar to bacterial SK protein and other ATP-utilizing enzymes (Pacold and Anderson, 1973; Huang et al., 1975; Schmidt et al., 1990). For this reason it has recently been postulated that SK may link to energy requiring shikimate pathway to the cellular energy balance (Maeda and Dudareva, 2012), however, direct experimental support for this hypothesis is currently lacking. In *Arabidopsis*, homologous genes named SKL1 and SKL2, which are functionally required for chloroplast biogenesis have been demonstrated to have arisen from SK gene duplication (Fucile et al., 2008). SKL1 and SKL2 orthologs have been found in several seed plant species, but not in green algae (Table 2).

## 5-*enol*ypyruvylshikimate 3-Phosphate Synthase

The 5-*enol*ypyruvylshikimate 3-phosphate synthase (EPSPS, 3-phosphoshikimate 1-carboxyvintltransferase) is the sixth step and here a second PEP is condensed with S3P to form 5-*enol*pyruvylshiukimate 3-phosphate (EPSP). Since EPSPS is the only known target for the herbicide glyphosate (Steinrucken and Amrhein, 1980), isoforms of this enzyme are often classified according to their sensitivity of glyphosate, glyphosate sensitive EPSPS class I is present in bacteria and plant species, whilst glyphosate insensitive EPSPS class II which has been reported in certain bacteria such as *Agrobacterium* (Fucile et al., 2011). In plants, different number of EPSPS isoforms is found in several species; only a single isoform in green algae, lycophytes, and bryophytes, but either one or two are found in monocot and dicot species (Table 2). Phylogenetic analysis of EPSPS genes revealed, atypically for genes associated with shikimate metabolism, that five major groups could be observed; (i) microphyte, (ii) bryophyte, (iii) Brassicaceae specific clade, (iv) monocot species, and (v) dicot species clade (Figure 2Ae). There are clear indications that duplicated EPSPS genes in *Arabidopsis*, apple, grapevine, soybean, and poplar are the result of independent duplication events within their lineages with both copies being maintained in *Arabidopsis* (Hamberger et al., 2006), however, the reason for the unique divergence in this gene of the pathway is currently unclear.

## Chorismate Synthase

Chorismate, the final product of the shikimate pathway, is subsequently formed by chorismate synthase (CS) which catalyzes the *trans*-1,4 elimination of phosphate from EPSP. CSs are categorized within one of two functional groups (i) fungal type bifunctional CS which are associated with NADPH-dependent flavin reductase or (ii) bacterial and plant type monofunctional CSs (Schaller et al., 1991; Maeda and Dudareva, 2012). The reaction catalyzed by CS requires flavin mononucleotide (FMN) and its overall reaction is redox neutral (Ramjee et al., 1991; Macheroux et al., 1999; Maclean and Ali, 2003). The FMN represents supplies an electron donor for EPSP which facilitates the cleavage of phosphate. The first cloned plant CS gene was that from *C. sempervirens* (Schaller et al., 1991) which contains a sole CS in its genome. Given that this gene has a 5′ plastid import signal sequence, these results indicate that there may be no CS outside of the plastid this species. Surveying other species revealed that one to two CS genes were present in green algae, lycophytes, and bryophytes as well as dicot specie but that one to three are present in the genomes of apple and leguminous species (Table 2). A phylogenetic analysis of CS genes reveals three major clades constituted by (i) microphyte, (ii) monocot, (iii) dicot species (Figure 2Af).

## Chorismate Mutase

Chorismate mutase catalyzes the first step of phenylalanine and tyrosine biosynthesis and additionally represents a key step of toward the branch split of tryptophan biosynthesis. CM catalyzes the transformation of chorismate to prephenate via a Claisen rearrangement. The bacterial minor CM proteins (AroQ type, class I CM) display monofunctional enzymatic activity whilst several bifunctional CMs such as CM-PDT, CM-PDH, and CM-DAHP have been additionally been found in fungi and bacteria (class II CM, Euverink et al., 1995; Romero et al., 1995; Chen et al., 2003; Baez-Viveros et al., 2004). In spite of the fact of only one CM gene is present in algae and lycophyte genomes, more a single gene copy (two to five) are found in bryophytes as well as monocot and dicot species (Table 2). In seed plants, the CM1 bears a putative plastid transit peptide, but CM2 does not and is additionally usually insensitive to allosteric regulation by aromatic amino acids (Benesova and Bode, 1992; Eberhard et al., 1996; Maeda and Dudareva, 2012). Several plant species, especially dicot plants, have an additional CM3 family gene which displays high sequence similarity to CM2 yet bears a putative plastid transit peptide. For example, *Arabidopsis* has three isozymes named AtCM1 (At3g29200), AtCM2 (At5g10870), and AtCM3 (At1g69370) (Mobley et al., 1999; Tzin and Galili, 2010). Phylogenetic analysis of the CS genes reveals three major clades constituting of (i) AtCM2 clade, (ii) microphyte and bryophyte clade, and (iii) AtCM2 clade (Figure 2Ba). Additionally, clade iii shows two sub-groups, (iii-a) AtCM3 sub-groups and (iii-b) AtCM1 sub-group (Figure 2Ba) (Eberhard et al., 1996). In spite of that the CM2 sub-group contains all species of seed plants, monocot species are not contained into AtCM3 sub-group. Recently the importance of CM has been extended beyond intracellular metabolism, In *Zea mays*, the chorismate mutase Cmu1 secreted by *Ustilago maydis*, a widespread pathogen characterized by the development of large plant tumors and commonly known as smut, is a virulence factor. The uptake of the Ustilago CMu1 protein by plant cells allows rerouting of plant metabolism and changes the metabolic status of these cells via metabolic priming (Djamei et al., 2011). It now appears that secreted CMs are found in many plant-related microbes and this form of host manipulation would appear to be a general weapon in the arsenal of plant pathogens.

## Prephenate Aminotransferase and Arogenate Dehydratase

Prephenate aminotransferase (PAT) and arogenate dehydratase (ADT) catalyze the final steps for production of phenylalanine. Whilst ADT was first cloned in 2007 (Cho et al., 2007; Huang et al., 2010), it is only more recently that PAT was cloned. Papers published in 2011 identified PAT in *Petunia hybrid*, *Arabidopsis thaliana*, and *Solanum lycopersicum* (Dal Cin et al., 2011; Maeda et al., 2011) and established that it directs carbon flux from prephenate to arogenate but also that it is strongly and co-ordinately upregulated with genes of primary metabolism and phenylalanine derived flavor volatiles. In plant species, a different number of PAT isoforms have been found. Although green algae only contain single PAT and ADT genes, monocot species have between one and two PATs and between two and four ADTs whilst dicot plants genomes contain the same number of PATs but two to eight ADTs (Table 2). Phylogenetic analysis of PAT genes shows three major clades of (i) microphyte, (ii) monocot, and (iii) dicot species (Figure 2Bb).

## Genes Involved in Plant Phenolic Secondary Metabolisms

Phenolic secondary metabolism displays an immense chemical diversity due to the evolution of enzymatic genes which are involved in the various biosynthetic and decorative pathways. Such variation is caused by diversity and redundancy of several key genes of phenolic secondary metabolism such as PKSs, cytochrome P450s (CYPs), Fe^2+^/2-oxoglutarate-dependent dioxygenases (2ODDs), and UDP-glycosyltransferases (UGTs). On the other hand, there are other general phenylpropanoid related biosynthetic genes, phenylalanine ammonia-lyase (PAL), cinnamate 4-hydroxylase (C4H), and 4-coumarate:coenzyme A ligase (4CL), which are required in order to differentiate various classes of phenolic secondary metabolism. All of these core genes encode important enzymes which activate a number of hydroxycinnamic acids to provide precursors for the biosynthesis of lignins, monolignals, and indeed all other major phenolic secondary metabolites in higher plants (Lozoya et al., 1988; Allina et al., 1998; Hu et al., 1998; Ehlting et al., 1999; Lindermayr et al., 2002; Hamberger and Hahlbrock, 2004). Since phenolic secondary metabolism display considerable species-specificity, investigation of the genes encoding the responsible biosynthetic enzymes are frequently used as an example of chemotaxonomy for understanding plant evolution. However, considering the evolution of these genes in isolation is rather restrictive a deeper understanding is provided by combining this with investigation of the evolution of the shikimate-phenylalanine biosynthetic genes in the green lineage.

## Conclusion

**Figure 3:**
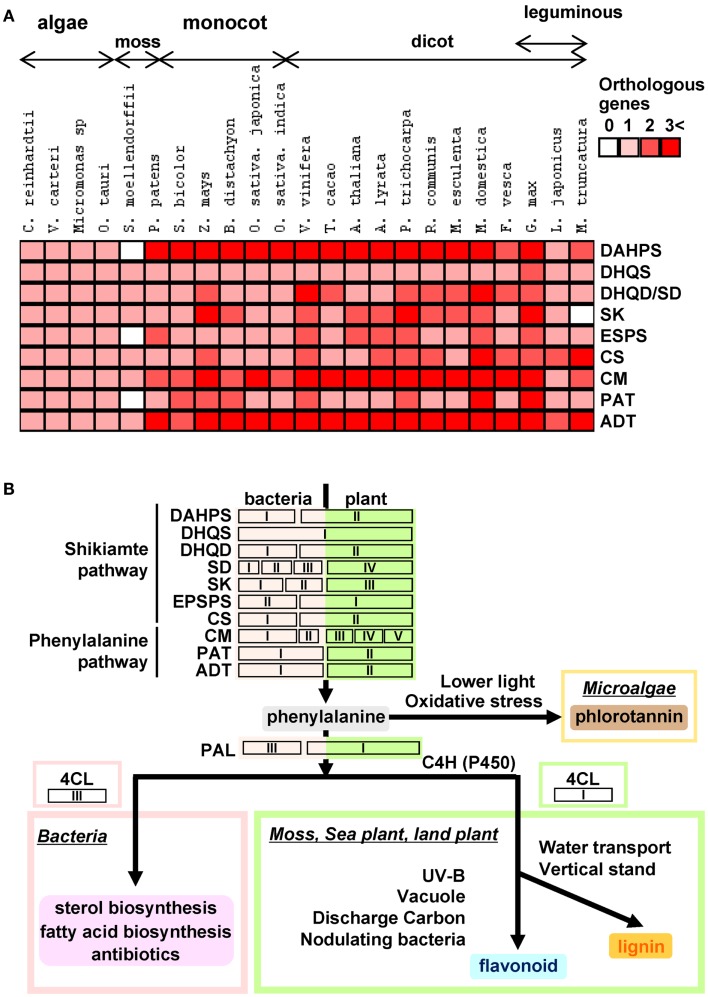
**Heat map for isoforms of shikimate-phenylalanine biosynthetic genes in plant genomes and hypothetical scheme for the evolution of phenylalanine derived phenolic secondary metabolism. (A)** Heap map overview of number of shikimate-phenylalanine biosynthetic gene isoforms in 25 species. **(B)** Hypothetical schematic figure for shikimate-phenylalanine biosynthetic genes and their evolution of phenolic secondary metabolism.

During the long evolutionary period covered from aquatic algae to land plants, plants have adapted to the environmental niches with the evolutionary strategies such as gene duplication and convergent evolution by the filtration of natural selection. Genes of plant shikimate biosynthesis have evolved accordingly (Figure 3). In this review, we demonstrated that biosynthetic genes of aromatic amino acid primary metabolism are well conserved between algae and all land plants. However, in contrast to algae species which have neither isoforms nor duplicated genes in their genomes, all land plants harbor gene duplications including tandem gene duplications which are particularly prominent in the cases of DAHPS, DHQD/SD, CS, CM, and ADT (Figure 3A; Table 2). Our phylogenetic analysis revealed clear separation between algae, monocots, dicots, woody species, and leguminous plants. Analysis of the presence and copy number of key genes across these species gives several hints as to how to improve our understanding of the scaffold from which these genes have evolved. However, the exact evolutionary pressures on genes of shikimate biosynthesis including the unique occurrence of the Arom complex will require considerable further studies. That said it is intriguing to compare and contrast biosynthetic genes of those downstream of them in the production of plant phenolics (Figure 3B). Interestingly, shikimate pathway genes are ubiquitous across the green lineage whilst this cannot be said for all downstream genes of phenylpropanoid biosynthesis. Furthermore, there is a much greater gene duplication within phenylpropanoid than shikimate biosynthesis (Figure 3A; Table 2). This fact also reflected in the level of chemical diversity of the respective pathways with the essentiality of the shikimate pathway preventing much diversity, but phenylpropanoid species often being redundant in function to one another. It would seem likely that the phenylpropanoid pathway initially arose via mutations accumulating in the shikimate pathway genes. However, whilst these were potentially beneficial in land plants for reasons we discuss in our recent review of these compounds (Tohge et al., 2013) they do not appear to share the essentiality of shikimate across the entire green lineage.

## Conflict of Interest Statement

The authors declare that the research was conducted in the absence of any commercial or financial relationships that could be construed as a potential conflict of interest.
